# Delayed Onset of Experimental Autoimmune Encephalomyelitis in Olig1 Deficient Mice

**DOI:** 10.1371/journal.pone.0013083

**Published:** 2010-09-29

**Authors:** Xiaoli Guo, Chikako Harada, Kazuhiko Namekata, Yoshinori Mitamura, Hiroshi Yoshida, Yoh Matsumoto, Takayuki Harada

**Affiliations:** 1 Department of Molecular Neurobiology, Tokyo Metropolitan Institute for Neuroscience, Tokyo, Japan; 2 Department of Ophthalmology, Institute of Health Biosciences, The University of Tokushima Graduate School, Tokushima, Japan; 3 Department of Neuro-ophthalmology, Tokyo Metropolitan Neurological Hospital, Tokyo, Japan; 4 Department of Molecular Neuropathology, Tokyo Metropolitan Institute for Neuroscience, Tokyo, Japan; Institut de la Vision, France

## Abstract

**Background:**

Olig1 is a basic helix-loop-helix (bHLH) transcription factor that is essential for oligodendrogenesis and efficient remyelination. However, its role in neurodegenerative disorders has not been well-elucidated.

**Methodology/Principal Findings:**

Here we investigated the effects of Olig1 deficiency on experimental autoimmune encephalomyelitis (EAE), an animal model of multiple sclerosis (MS). We show that the mean disease onset of myelin oligodendrocyte glycoprotein (MOG)-induced EAE in Olig1^−/−^ mice is significantly slower than wide-type (WT) mice (19.8±2.2 in Olig1^−/−^ mice and 9.5±0.3 days in WT mice). In addition, 10% of Olig1^−/−^ mice did not develop EAE by the end of the observation periods (60 days). The severity of EAE, the extent of demyelination, and the activation of microglial cells and astrocytes in spinal cords, were significantly milder in Olig1^−/−^ mice compared with WT mice in the early stage. Moreover, the visual function, as assessed by the second-kernel of multifocal electroretinograms, was better preserved, and the number of degenerating axons in the optic nerve was significantly reduced in Olig1^−/−^ mice. Interestingly, Olig1 deficiency had no effect on T cell response capability, however, it reduced the expression of myelin proteins such as MOG, myelin basic protein (MBP) and myelin-associated glycoprotein (MAG). The expression of Olig2 remained unchanged in the optic nerve and brain, and it was reduced in the spinal cord of Olig1^−/−^ mice.

**Conclusions/Significance:**

Our results suggest that the Olig1 signaling pathways may be involved in the incidence rate and the severity of neurological symptoms in MS.

## Introduction

Multiple sclerosis (MS) is an inflammatory disease of the central nervous system (CNS) characterized by progressive immune-mediated destruction of the myelin sheath. The inflammatory process is thought to be mediated in part by T lymphocytes and microglia/macrophage that are recruited to the CNS in response to chemotactic signals [1]. In MS, remyelination is an inconsistent event though there are substantial numbers of oligodendrocyte progenitor cells (OPCs) observed in the lesion sites [2]–[5]. Thus, understanding the molecular mechanisms of remyelination and the factors related to the limitations of remyelination is particularly important to identify potential therapeutic targets for repair. Recent studies have demonstrated that some developmental pathways that restrict oligodendrocyte maturation, such as signaling mediated by Notch and Lingo-1, are re-expressed during MS [6]–[8]. One important complication in MS is optic neuritis, which is an acute inflammatory demyelinating syndrome of the CNS. Since it can cause severe visual loss that is currently irreversible, especially in the optic-spinal form of MS or neuromyelitis optica (NMO) [9], [10], it draws much attention to finding a treatment that will restore the visual function.

Olig1 is a basic helix-loop-helix (bHLH) transcription factor expressed in mature oligodendrocytes, the myelinating cells of the CNS, and their progenitor cells in the developing CNS [11], [12]. Targeted disruption of Olig1 indicated that Olig1 plays important roles during the development and maturation of oligodendrocytes [13]. Olig1 is localized in the nucleus during the development phase and may function as a transcriptional regulator for myelin-specific gene expression such as myelin oligodendrocyte glycoprotein (MOG), myelin basic protein (MBP) and myelin-associated glycoprotein (MAG) [12], [14]. In addition, Olig1 might be required for remyelination in the adult brain and spinal cord [12]. Indeed, a previous study using toxin-induced demyelination models in Olig1^−/−^ mice demonstrated that Olig1 function is essential for the remyelination phase of both cuprizone- and lysolecithin-induced demyelination in brain and spinal cord, respectively [13]. These results suggest a critical role for Olig1 in the repair of the adult CNS, however, its biological functions are not fully understood.

In the present study, we investigated the effects of Olig1 deficiency on experimental autoimmune encephalomyelitis (EAE), an animal model of MS. There are two Oligl^−/−^ mouse lines available currently, one with a *PGKneo* cassette and the other with this cassette removed [12], [14]. Mice with the *PCKneo* cassette are viable while mice without the *PGKneo* cassette die prematurely at around postnatal day (P) 14, or they do not survive beyond P17 [14]. It has been suggested that the presence of *PGKneo* in the Olig1 locus may induce increased Olig2 expression that compensates for the Olig1 deficiency and thus, allowing the mice to survive. However, the reason for this discrepancy in these two Oligl^−/−^ mouse lines is still unclear. In the present study, we have used Oligl^−/−^ mice with the *PGKneo* cassette. Considering the delayed remyelination in toxin-induced demyelination models [13], we expected that the severity of EAE in Olig1^−/−^ mice might be greater than WT mice. However, our present study revealed a delayed onset of EAE and mild neurological symptoms in Olig1^−/−^ mice. We also examined the visual function by multifocal electroretinograms, a non-invasive method, in order to provide an insight into the pathology of optic neuritis, which is often associated with MS/EAE.

## Results

### Effect of Olig1 deficiency on EAE disease onset, incidence and severity

**Figure 1:**
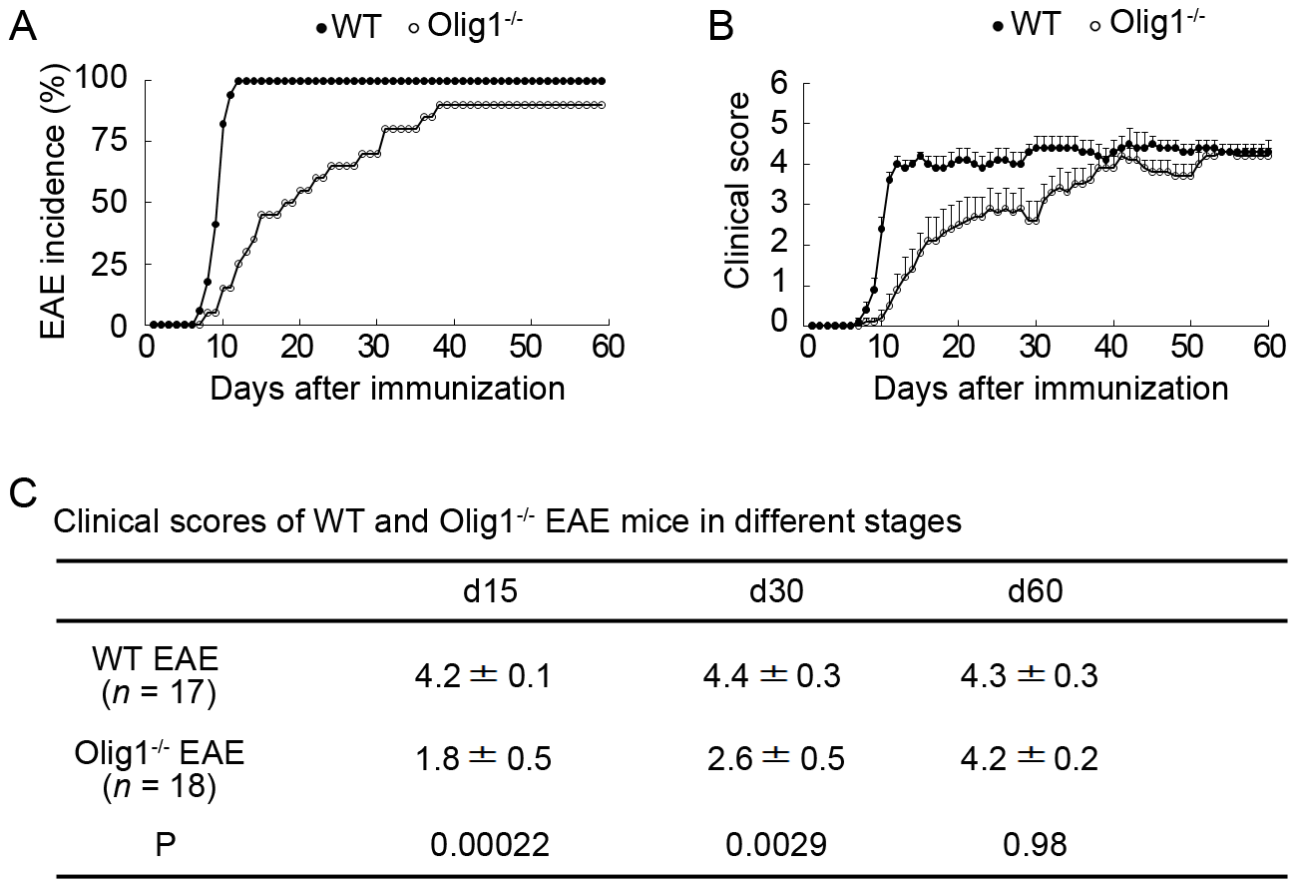
Delayed EAE disease onset in Olig1^−/−^ mice. (A) EAE disease incidence in wild-type (WT; *n* = 17) and Olig1^−/−^ (*n* = 20) mice. (B) Clinical evaluation of WT and Olig1^−/−^ EAE mice during a period of 60 days after MOG immunization. (C) Clinical scores of WT and Olig1^−/−^ EAE mice in different stages.

The mice were observed for a period of 60 days after MOG immunization. As shown in Figure 1A, wild-type (WT) mice started to show EAE disease signs 7 days after disease induction, and reached an incidence of 100% in 9.5±0.3 days (*n* = 17). However, a significant delay of disease onset was found in Olig1^−/−^ EAE mice with a mean disease onset day of 19.8±2.2 (*n* = 18, *P*<0.001). More interestingly, 10% of Olig1^−/−^ mice did not develop EAE by the end of the observation period. Daily clinical scoring revealed that the severity of EAE was significantly milder in Olig1^−/−^ mice compared with WT mice before day 50, except for the period between days 36–44 (Figure 1B). However, the severity of Olig1^−/−^ EAE mice became comparable with WT EAE mice by the end of the experiment (Figure 1B). The clinical scores of Olig1^−/−^ EAE mice at various time points were summarized in Figure 1C.

**Figure 2:**
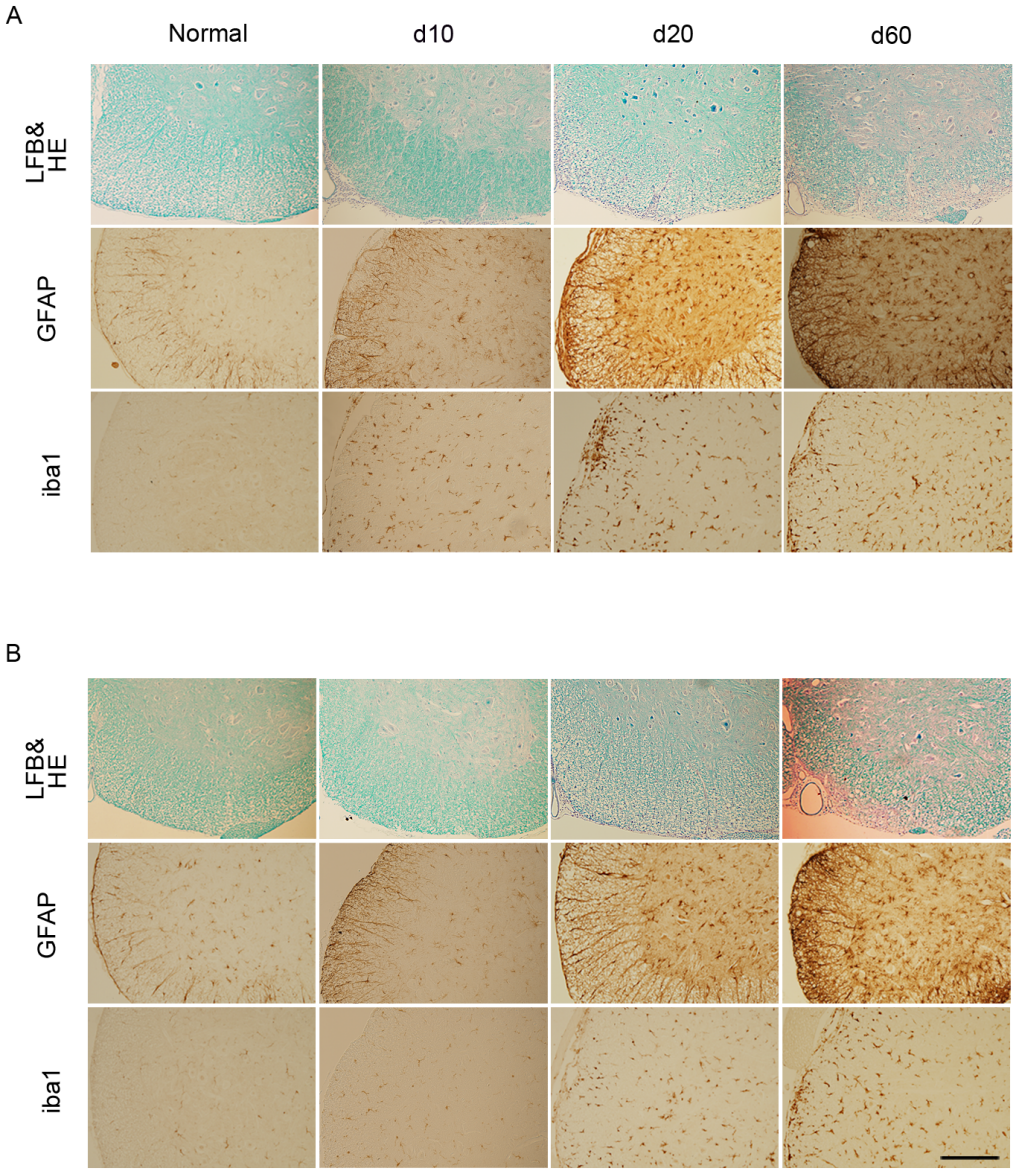
Histopathology of the spinal cords in EAE mice. Representative histology of the spinal cords of WT (A) and Olig1^−/−^ mice (B) in d10, d20 and d60. Lumbar spinal cords were stained with luxol fast blue (LFB) and hematoxylin and eosin (HE) (upper panels) and either an anti-GFAP (middle panels) or anti-iba1 antibody (lower panels). Scale bar: 200 µm and applies to all panels.

**Figure 3:**
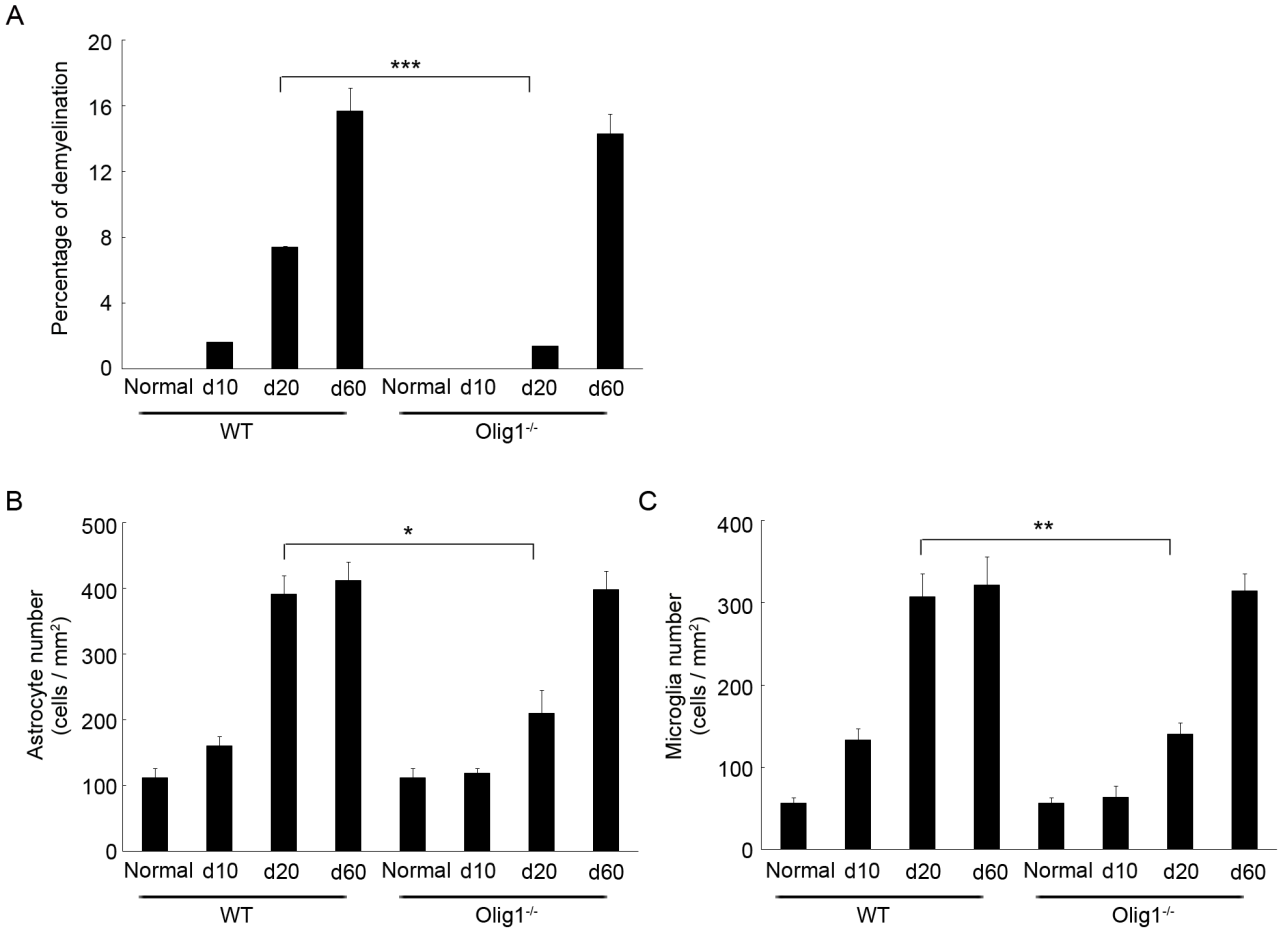
Quantification of histopathology of the spinal cords in EAE mice. Quantitative analysis of the extent of demyelination (A), GFAP-positive (B) and iba1-positive (C) cells in the spinal cord. The areas of demyelinated regions in the white matter were measured by ImageJ 1.43u and expressed as a percentage of the whole area of the white matter. GFAP- and iba1-positive cells were counted per unit area (0.143 mm^2^) in the middle region of the ventral horn. ***P<0.001; **P<0.01; *P<0.05.

We then examined the spinal cords histopathologically at different time points (Figure 2). Numerous cell infiltrates were observed in the spinal cord of the WT EAE mice on day 10, while almost no cell infiltrates were found in Olig1^−/−^ EAE mice at this time point (upper panels in Figure 2A and 2B). Furthermore, on day 20, demyelination and cell infiltrates were both obvious in the spinal cords of the EAE mice (upper panels in Figure 2A, 2B and 3A), which were much milder in Olig1^−/−^ mice compared with WT mice. However, on day 60, the extents of demyelination and cell infiltrate were comparable between the two genotypes (upper panels in Figure 2A, 2B and 3A). Since EAE induction also induces the activation of astrocytes and microglia [15], we next investigated the effect of Olig1 deficiency on the extent of neuroinflammation. Both astrocyte and microglia remained inactivated on day10 in Olig1^−/−^ EAE mice, and when activated, their activation were milder compared with WT EAE mice on day 20 (middle and lower panels in Figure 2A, 2B and 3B, 3C). This difference between the two genotypes diminished again on day 60.

**Figure 4:**
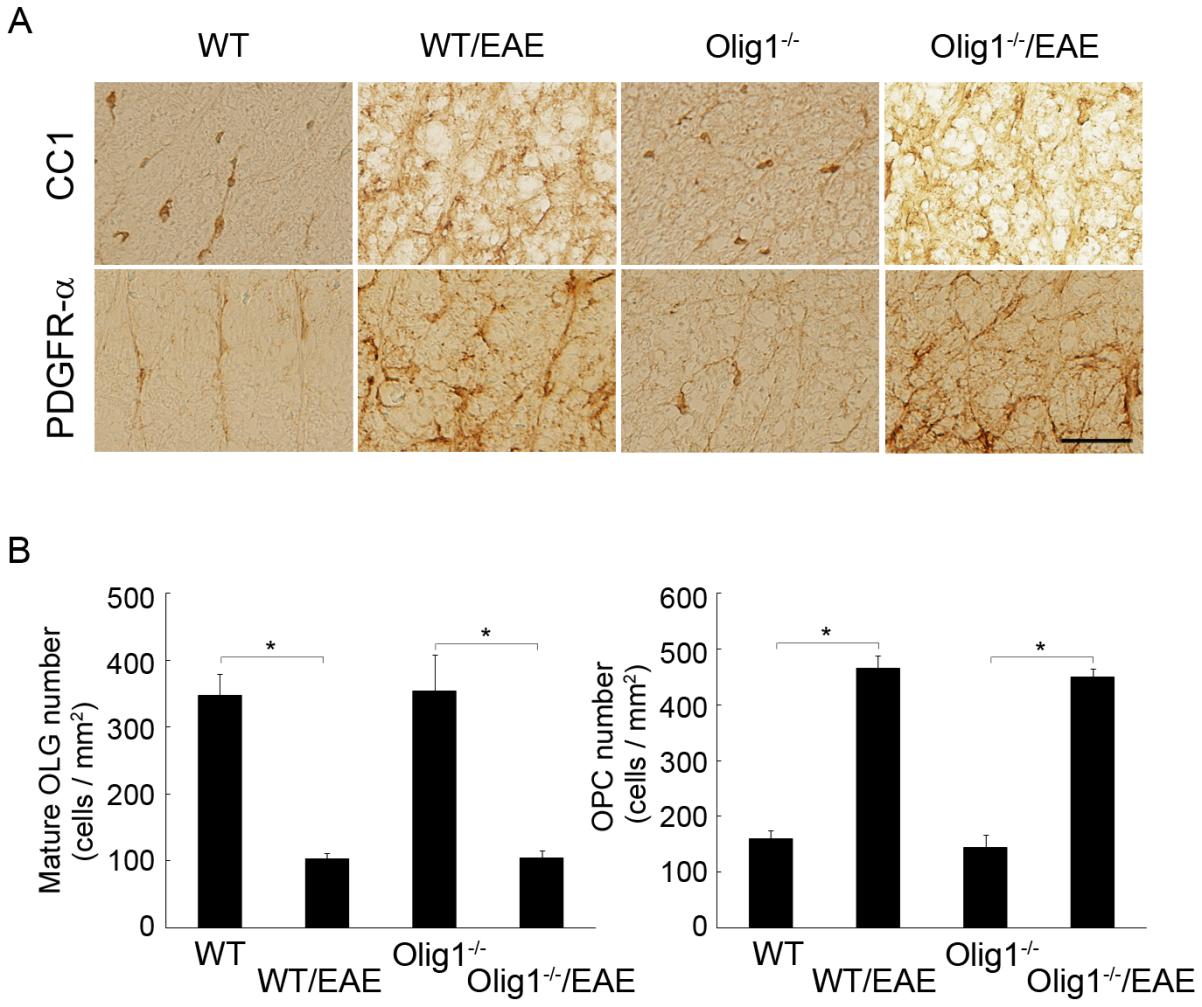
The effect of Olig1 deficiency on the number of oligodendrocytes and their progenitor cells during EAE. (A) Representative immunostaining of CC1 and PDGFR-α in the white matter of the spinal cord. Scale bar: 40 µm and applies to all panels. (B) Quantitative analysis of mature oligodendrocytes and their progenitor cells. *P<0.001. OLG, oligodendrocyte; OPC, oligodendrocyte progenitor cell.

We also performed immunohistochemical analysis to determine whether Olig1 deficiency affects the number of oligodendrocytes and oligodendrocyte progenitor cells (OPCs) in the white matter of the spinal cord during EAE. The number of CC1-positive mature oligodendrocytes was clearly reduced in both of WT and Olig1^−/−^ EAE mice on d60 after MOG-immunization (upper panels in Figure 4A and 4B). No significant difference was observed between the two genotypes (Figure 4B). On the other hand, the number of OPCs, as demonstrated with PDGFR-α staining, was significantly increased upon EAE induction (lower panels in Figure 4A and 4B). However, there was also no difference between the two genotypes (Figure 4B).

### Effect of Olig1 deficiency on optic neuritis

**Figure 5:**
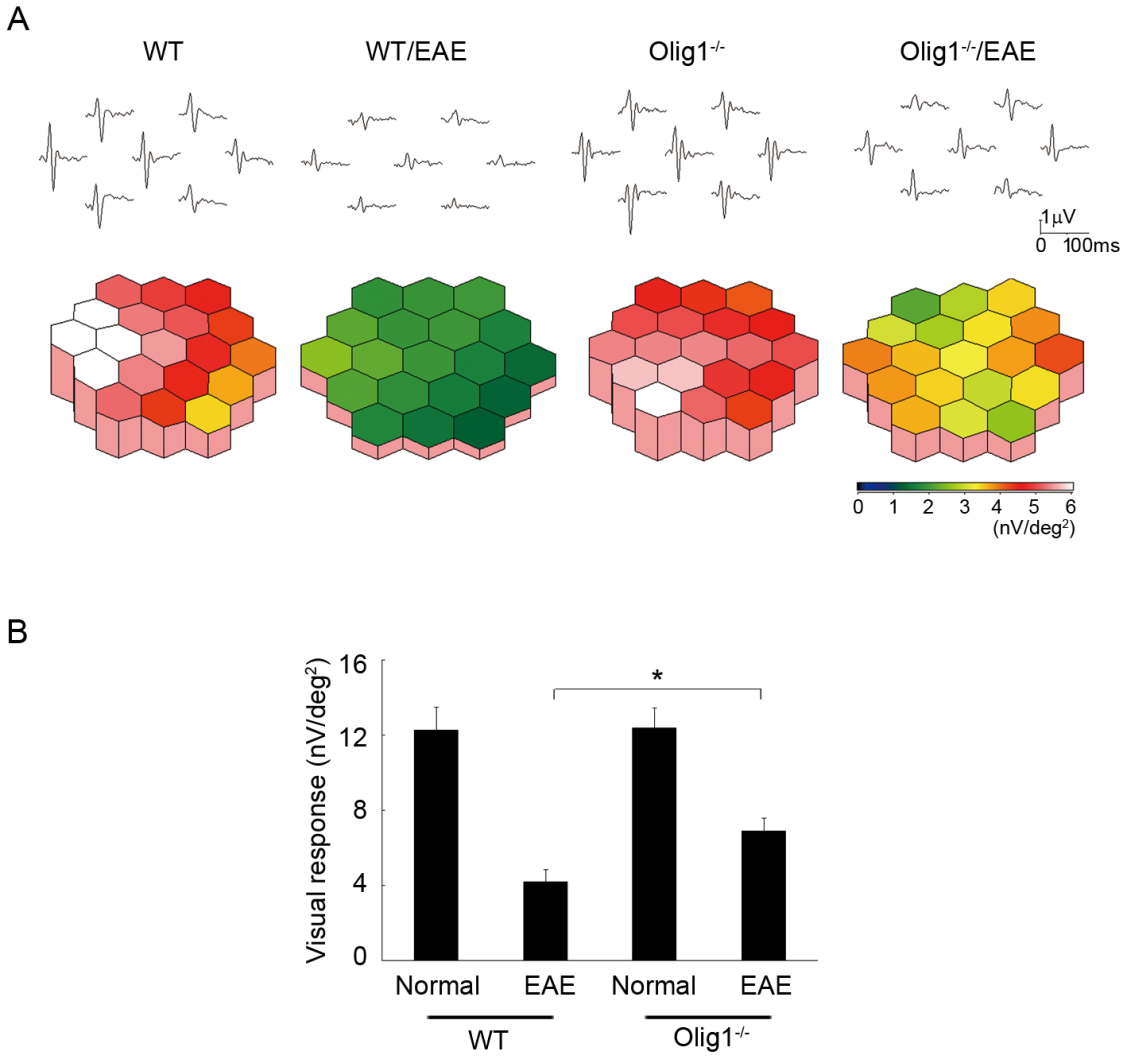
Attenuated impaired visual function in Olig1^−/−^ EAE mice. (A) The averaged visual responses from six WT and Olig1^−/−^ mice were examined by multifocal electroretinograms (mfERGs). The visual stimulus was applied to seven different areas in the retina. The seven individual traces demonstrate the average responses to the visual stimulus at the corresponding stimulus area (upper panels). The degree of retinal function is presented in the color bar. The higher score (red) indicates highly sensitive visual function and lower score (green) indicates retinal dysfunction. Values are given in nV per square degree (nV/deg^2^). (B) Quantitative analysis of the visual response amplitude. The sum of the response amplitudes for each stimulus element was divided by the total area of the visual stimulus. *P<0.05.

As MS often induces visual disturbance, especially in the optic-spinal form of MS or neuromyelitis optica (NMO) [9], [10], we next examined the effect of Olig1 deficiency on the severity of optic neuritis from both the physiological and histopathological aspects. To this aim, we first investigated the visual functions of EAE mice using multifocal electroretinograms (mfERGs), an established noninvasive method for effectively measuring visual function [16], [17]. The response topography demonstrated that the visual function of WT EAE mice was impaired in all visual fields, but it was clearly better preserved in Olig1^−/−^ EAE mice (Figure 5A). Furthermore, quantitative analysis revealed that the visual function in Olig1^−/−^ EAE mice (6.91±0.69 nV/deg^2^; *n* = 6) was significantly better than WT EAE mice (4.19±0.70 nV/deg^2^; *n* = 6) (*P* = 0.032, Figure 5B).

**Figure 6:**
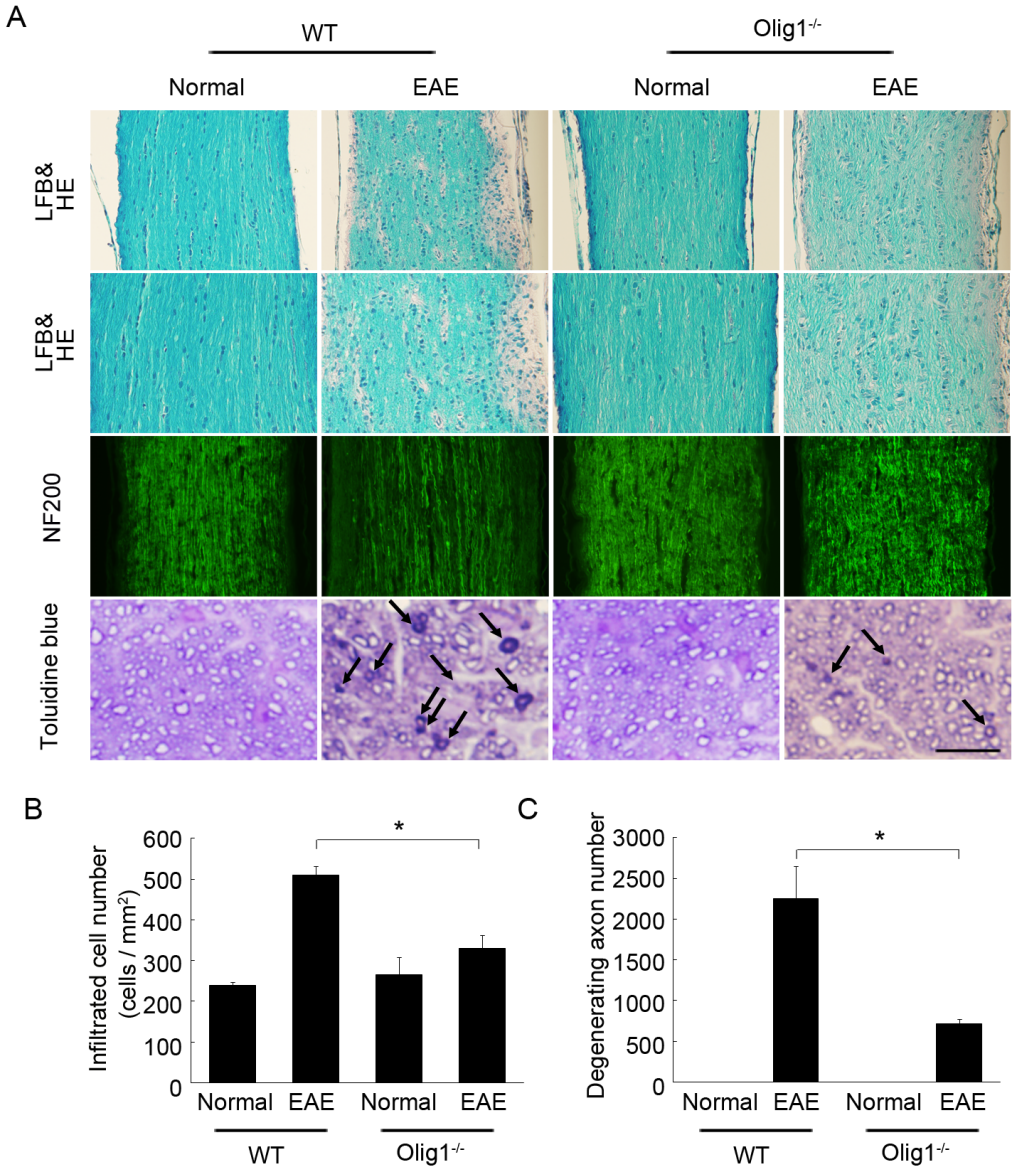
Representative histopathology of the optic nerves in EAE mice. (A) Optic nerves were stained with luxol fast blue (LFB) and hematoxylin and eosin (HE) (upper two panels) or NF200 and toluidine blue for the semithin transverse sections (lower two panels). The arrows point to the degenerating axons. Scale bar: 100 µm for the first and third panels, 75 µm for the second panels and 15 µm for the lower panels. (B) Quantitative analysis of cell infiltrates in the longitudinal section of the optic nerve. (C) Quantitative analysis of degenerating axons in the transverse section of the optic nerve. *P<0.01.

Histopathological analysis revealed that in WT EAE mice, numerous inflammatory cells infiltrated into the optic nerve lesion (upper two panels in Figure 6A). However, the number of cell infiltrates was significantly reduced in Olig1^−/−^ EAE mice (Figure 6B). In addition, the extent of demyelination was clearly mild in Olig1^−/−^ EAE mice. To further analyze morphological changes in the optic nerve, NF200-stained sections and semithin transverse sections were observed under a microscope. The level of NF200 staining for intact axons was largely reduced (third panels in Figure 6A), indicating severe nerve fiber damage in WT EAE mice. In contrast, the NF200 staining in Olig1^−/−^ EAE mice was more intense than WT EAE mice (third panels in Figure 6A), indicating a higher rate of surviving axons in Olig1^−/−^ EAE mice. Moreover, the degenerating axons had abnormally dark axonal profiles and the density of axons through the optic nerve was declined in WT EAE mice, but the number of degenerating axons was clearly reduced in Olig1^−/−^ EAE mice (lower panels in Figure 6A, 6C). Taken together, these data demonstrate that Olig1 deficiency attenuates both the histological and functional aspects of EAE-induced optic neuritis.

### Reduced MOG expression in Olig1 deficient mice

**Figure 7:**
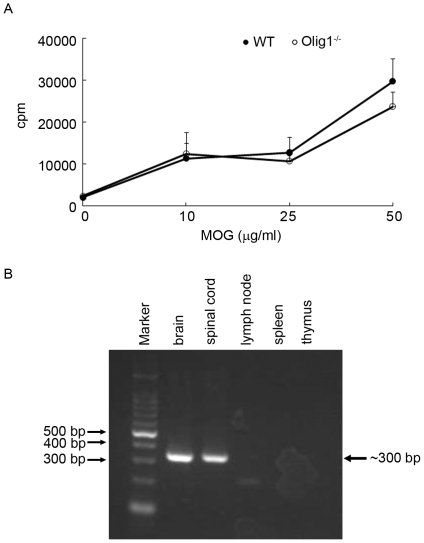
The effect of Olig1 deficiency on T cell proliferation capability. (A) Proliferative responses of MOG-specific T cells isolated from WT and Olig1^−/−^ EAE mice (*n* = 4). (B) RT-PCR analysis of Olig1 expression in brain, spinal cord, lymph node, spleen and thymus of WT mice.

We then investigated the mechanisms leading to the delayed onset of EAE and attenuated optic neuritis in Olig1^−/−^ mice. Since EAE is a T cell-mediated autoimmune disease, we first assessed the effect of Olig1 deficiency on T-cell proliferation capability. Freshly isolated T cells from WT and Olig1^−/−^ MOG-immunized mice were stimulated with different concentrations of MOG. However, no significant difference of T cell proliferation response was found between WT and Olig1^−/−^ mice (Figure 7A). We also examined whether Olig1 was expressed in the major immune organs. RT-PCR analysis revealed strong expression of Olig1 mRNA in the spinal cord and brain (Figure 7B). However, no mRNA expression was found in the lymph node, spleen and thymus (Figure 7B), which further suggests that delayed disease onset of Olig1^−/−^ EAE mice was independent of T cell response.

**Figure 8:**
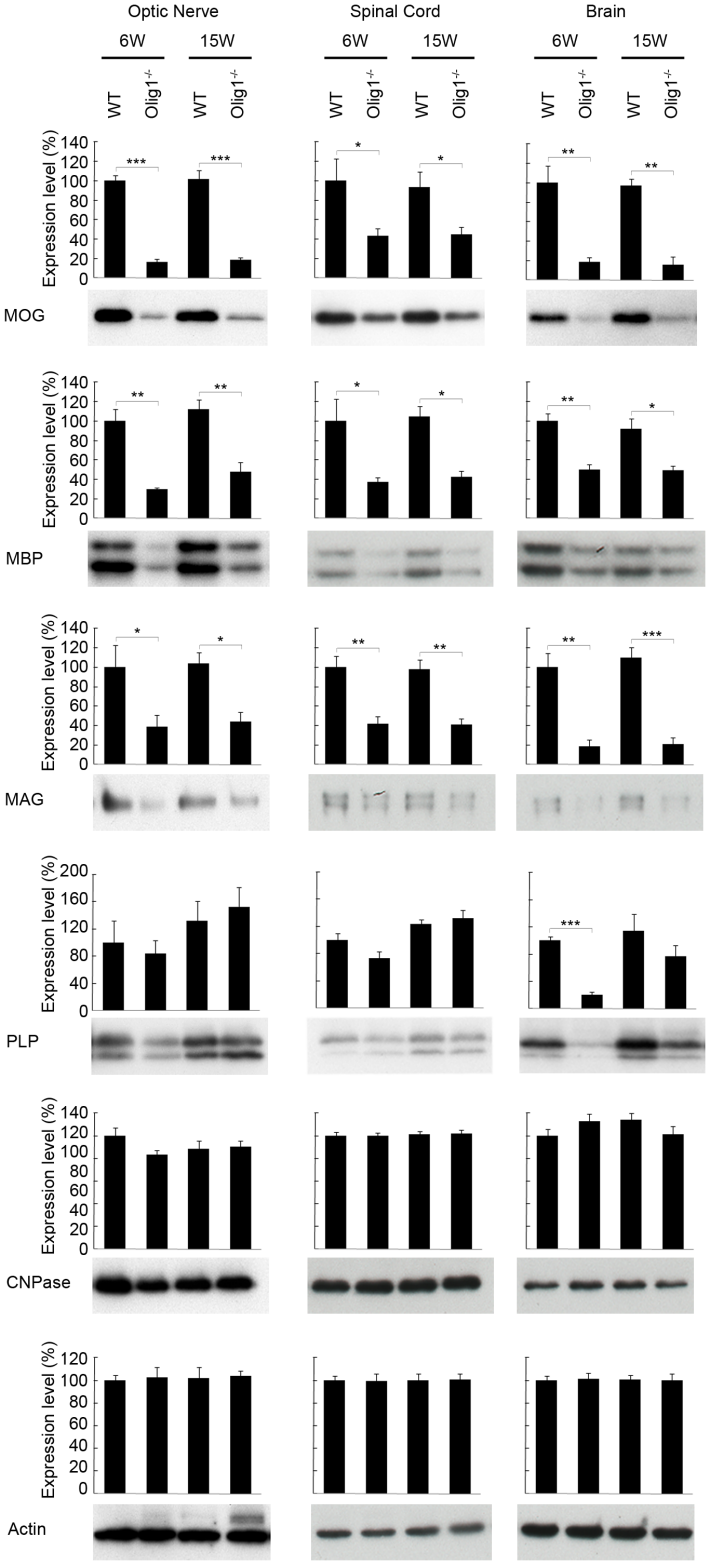
Myelin protein expression levels in Olig1^−/−^ mice. The expression of MOG, MBP, MAG, PLP and CNPase in the tissues was investigated by Western blotting. Tissues were isolated from 6 week- and 15 week-old WT and Olig1^−/−^ mice. ***P<0.001; **P<0.01; *P<0.05.

Olig1 may function as a transcriptional regulator for myelin-specific gene expression. Interestingly, transcriptome profile analysis of optic nerves from Olig1^−/−^ mice without the *PGKneo* cassette revealed a large amount of reductions of some myelin protein transcripts including MBP, MAG and MOG [13], [18]. We then investigated the expression of myelin proteins in Olig1^−/−^ mice with the *PGKneo* cassette [12], which are the mouse line used in the present study. Western blotting analysis was carried out on tissues including the optic nerve, spinal cord and brain, which were isolated from non-EAE 6 week- (the age when EAE was induced) and 15 week- (the age when EAE mice were sacrificed) old mice (Figure 8). Among the myelin proteins investigated, expression levels of MOG, MBP and MAG, except that of CNPase, were significantly attenuated in 6 week-old Olig1^−/−^ mice compared with age-matched WT mice. In addition, a similar trend was also detected in 15 week-old Olig1^−/−^ mice compared with WT mice. One intriguing point is that the expression level of PLP was attenuated significantly in the brain, but not in the optic nerve and spinal cord of 6 week-old Olig1^−/−^ mice. Moreover, the expression level of PLP in the brain became comparable between 15 week-old WT and Olig1^−/−^ mice, which indicated that the process of PLP expression to reach its optimal level might be delayed in the Olig^−/−^ mice.

**Figure 9:**
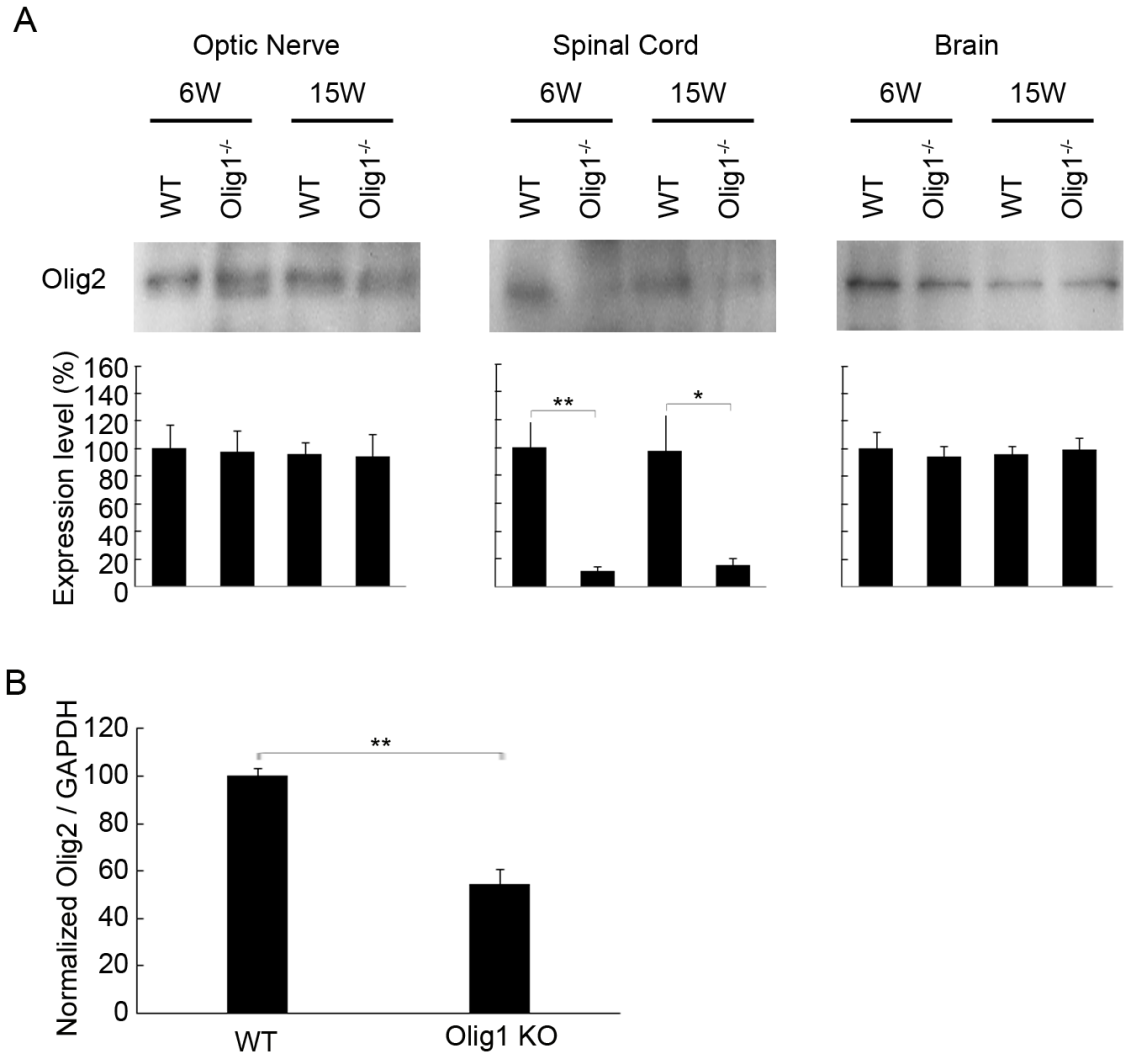
Olig2 expression levels in Olig1^−/−^ mice. (A) The expression of Olig2 in the tissues was investigated by Western blotting. Tissues were isolated from 6 week- and 15 week-old WT and Olig1^−/−^ mice. (B) Quantitative real-time PCR analysis of Olig2 mRNA in the spinal cord. **P<0.01; *P<0.05.

As already mentioned earlier, the reason for survival of the Olig1^−/−^ mice with the *PGKneo* cassette, as opposed to the mice without it, has been speculated to be due to the compensatory mechanisms induced by increased Olig2 expression [12]. Considering the significance for confirming this speculation, it is surprising that Olig2 expression in Olig^−/−^ mice has never been reported in the literature so far. Hence, we have studied Olig2 expression levels in the tissues isolated from 6 and 15 week-old WT and Olig1^−/−^ mice with the *PGKneo* cassette. Interestingly, on the contrary to what had been suggested, the Olig2 protein expression remained unchanged in the optic nerve and brain, while it was significantly reduced in the spinal cord of Olig1^−/−^ mice (Figure 9A). Quantitative real-time PCR analysis of Olig2 in the spinal cord further confirmed this attenuation (Figure 9B).

## Discussion

Olig1 is an important transcript factor expressed in mature oligodendrocytes and their progenitor cells, however, the biological functions of Olig1 are not fully elucidated. In the present study, we examined the effect of Olig1 deficiency on EAE and optic neuritis, which is one of the presenting signs of MS. We hypothesized that the clinical signs of Olig1^−/−^ EAE mice might be more severe than WT EAE mice based on the results from the toxin-induced demyelination models [13]. However, Olig1^−/−^ mice displayed delayed onset of EAE, and the clinical signs of Olig1^−/−^ EAE mice became comparable with WT EAE mice only in the late phase. Moreover, in contrast to the previous assumption [13], the numbers of mature oligodendrocytes and OPCs were not decreased in Olig1^−/−^ EAE mice. The remyelination process in EAE models is much less obvious compared with the chemical models of toxin-induced demyelination, which may have hampered the detection of subtle changes in the number of oligodendrocytes and OPCs. Furthermore, Olig1 deficiency attenuated optic neuritis induced by EAE. Since T cell response was unaffected by Olig1 deficiency, we speculate that reduced MOG expression in the spinal cord and optic nerve might contribute to the observed phenotype of Olig1^−/−^ EAE mice.

The pathogenic changes underlying autoimmune disorders are thought to be initiated and driven by T cell populations that recognize and respond to autoantigens [19]. In a study to demonstrate the immunological basis for MOG, MOG deficient (MOG^−/−^) mice exhibited no clinical or histological abnormalities when immunized with recombinant MOG, or with the MOG 35–55 peptide sequence [20]. Moreover, although MOG accounts for only about 0.1% of total CNS myelin protein, MOG^−/−^ mice showed a delayed onset of EAE when immunized with whole myelin and the clinical signs were significantly milder compared with WT mice, suggesting that anti-MOG response is the major pathogenic component of the autoimmune response directed against myelin. Thus, it seems to be logical that reduced MOG expression in the Olig1^−/−^ mice might largely contribute to the delayed onset of EAE. On the other hand, multiple antigen recognition exists in MS. In a recent study to explore the role of the autoantigen in spontaneous autoimmunity, a transgenic mouse expressing a MOG-specific T cell receptor was investigated [19]. As predicted, the mice developed spontaneous EAE in the presence of MOG, but they also developed EAE in the absence of MOG due to recognition of a non-MOG autoantigen corresponding to an epitope on neurofilament-M. The authors refer to the recognition of multiple independent autoantigens by a single T cell population as cumulative autoimmunity and suggest that these additive responses contribute to particularly vigorous immune reactions and may play a role in spontaneously developing autoimmune disorders in humans. Therefore, the reduced expressions of MBP and MAG in Olig1^−/−^ mice might also contribute to the delayed onset of EAE. One point to note is that the CNPase and PLP expressions in the optic nerve and spinal cord remained unchanged although the expressions of MOG, MBP and MAG were all reduced in Olig1^−/−^ mice, which might be explained by the possibility that the CNPase and PLP expressions are only weakly regulated by Olig1.

Since the expression levels of MOG, MAG and MBP were reduced (Figure 8), one might expect that there is less myelination in Olig1^−/−^ axons. However, there was no difference in the structure of the myelin in the spinal cord and optic nerve between non-EAE Olig1^−/−^ mutants and WT mice (Figure 2 and 6). These findings are consistent with the initial report on the characterization of these mutant mice, in which it demonstrated this mouse line undergoes normal development [12]. There is a hypothesis that increased Olig2 expression compensates for the Olig1 deficiency. However, our data demonstrated that Olig2 expression remained unchanged in the optic nerve and brain, and in fact, it attenuated in the spinal cord in Olig1^−/−^ mice (Figure 9). It will be intriguing to investigate other candidate genes that may be responsible for the survival of this Olig1^−/−^ mouse line. One explanation for the unaltered myelin formation despite the altered level of MOG, MAG and MBP might be that the extent of reduction, which is subtle compared with the large reductions observed in the mice without the *PGKneo* cassette [18], was not enough to induce a significant change in the myelin formation during development. This speculation can be supported by the observation that not all myelin protein levels were affected; we have shown in this study that the expression levels of CNPase and PLP were unchanged in non-EAE adult Olig1^−/−^ mice (Figure 8). If there was in fact less myelination in these mice, it would be logical to expect that they should be more susceptible to EAE induction. However, on the contrary, our results demonstrated that these mice had reduced EAE severity in the early phase of the experiment (Figure 1B). Hence, reduced expressions of MOG, MBP and MAG did not impair myelin formation during development, but contributed to alleviate EAE signs in Olig1^−/−^ mice.

Genetic ablation of some genes has been reported to have significant effects on the disease onset of EAE. For example, MyD88^−/−^ mice were reported to be completely EAE resistant [21]. In addition, TLR9^−/−^ and TNF^−/−^ mice demonstrated a significant delay in EAE disease onset relative to WT mice [21], [22]. However, all of these onset delays were derived from the effects of the genetic ablation on the response of dendritic cells or T cells, which is different from the effects of Olig1 deficiency on myelin protein expressions observed in the present study. Thus, our results may have importance in the path to searching for factors responsible for the pathogenesis of MS other than such cell responses in the immune system.

In the present study, the results from the second-order kernel of mfERGs, which is a sensitive indicator of visual function [16], [17], demonstrated that the degree of visual impairment in Olig1^−/−^ EAE mice *in vivo* was less than WT EAE mice. Currently, corticosteroid is the only treatment option available for acute demyelinating optic neuritis, but future therapies could include downregulation of Olig1 as well as use of neuroprotective agents [16], [23]. The study by Arnett et al. demonstrated that Olig1 function is essential for the remyelination phase of both cuprizone- and lysolecithin-induced demyelination in brain and spinal cord, respectively [13]. Combining these data with our data, one may speculate that downregulation of Olig1 during inflammation and upregulation of Olig1 in the remyelination phase may prove to be one of the useful strategies against demyelinating diseases.

In addition to the expression in oligodendrocytes, Olig1 has also been demonstrated to be expressed in a subset of astrocytes [24]. Since GFAP expression was significantly upregulated in the spinal cord of EAE mice and chemokine released from astrocytes was implicated in the pathogenesis of EAE, it will be intriguing to elucidate the possible effects of Olig1 in astrocytes on microglia cell responses during EAE. On the other hand, it was hypothesized that genetic variants in the Olig1 gene might influence remyelinating capacity and vulnerability to the consequences of a demyelinating event [25]. Although some coding variants in the Olig1 gene have been identified in patients with MS [26], there is still no evidence to support this hypothesis. In the present study, we demonstrate that Olig1 deficiency, or reduced expression of MOG, MBP and MAG caused by Olig1 deficiency, delayed the onset of EAE. Thus, Olig1 signaling pathways may be involved in the incidence rate and the severity of neurological symptoms in MS. Modulation of Olig1 and myelin protein expression levels may provide novel therapeutic opportunities for MS.

## Materials and Methods

### Ethics Statement

All mice experiments were approved by the Tokyo Metropolitan Institute for Neuroscience Committee for Animal Research (permit number; 22-01/institutional review board; Dr. Norio Ishizuka).

### Mice

Olig1^−/−^ mice and the control WT mice are on the same C57BL/6J background. Olig1^−/−^ mice were generated as described [12]. Female WT and Olig1^−/−^ mice were maintained at the animal facilities of the Tokyo Metropolitan Institute for Neuroscience and were 6–8 weeks of age at the time of immunization.

### Induction and assessment of EAE

EAE was induced with rat myelin oligodendrocyte glycoprotein (MOG)_35–55_ peptide (MEVGWYRSPFSRVVHLYRNGK) as previously reported [15], [16]. Briefly, mice were subcutaneously injected with 100 µg of MOG_35–55_ mixed with 500 µg of heat-killed *Mycobacterium tuberculosis* H37RA (Difco, Detroit, MI, USA) emulsified in complete Freund's adjuvant. Each mouse also received intraperitoneal injections of 500 ng pertussis toxin (Seikagaku, Tokyo, Japan) immediately and 48 h after the immunization. Clinical signs were scored daily as follows: 0, no clinical signs; 1, loss of tail tonicity; 2, flaccid tail; 3, impairment of righting reflex; 4, partial hind limb paralysis; 5, complete hind limb paralysis; 6, partial body paralysis; 7, partial forelimb paralysis; 8, complete forelimb paralysis or moribund; 9, death.

### Histopathology and immunohistochemistry

Optic nerves and spinal cords were examined on day 10, day 20 and day 60 after MOG immunization as previously reported [15], [16]. Sections were stained with luxol fast blue (LFB) followed by hematoxylin and eosin (HE). Transversal semithin (500 nm) sections of optic nerve were stained with toluidine blue [17]. Immunohistochemistry was performed using the following primary antibodies: rabbit anti-iba1 (1.0 µg/ml) [27], mouse anti-GFAP (50 µg/ml; Progen, Toowong, Australia), mouse anti-CC-1 (1∶100; Calbiochem, Gibbstown, NJ, USA), rabbit anti-PDGFR-α (1∶200; Santa Cruz, Santa Cruz, CA, USA) and mouse anti-NF200 (1∶400; Sigma, St. Louis, MO, USA). Quantitative analysis of immunopositive cell number or stained region was carried out using NIH Image (ImageJ 1.43u).

### Multifocal electroretinograms (mfERGs)

mfERGs were measured in various time points after MOG immunization as previously reported [16], [17]. Briefly, mice were anesthetized by an intraperitoneal injection of a mixture of xylazine (10 mg/kg) and ketamine (25 mg/kg). Pupils were dilated with 0.5% phenylephrine hydrochloride and 0.5% tropicamide. mfERGs were recorded using a VERIS 6.0 system (Electro-Diagnostic Imaging, Redwood City, CA, USA).

### Proliferation assay of T cells

Proliferative responses of lymph node (LN) cells from WT and Olig1^−/−^ mice on day 9 after MOG immunization were assayed in microtiter wells by uptake of [^3^H]thymidine as previously reported [15]. LN cells (5×10^5^ cells/well) were cultured with given concentrations of MOG_35–55_ for 3 days, with the last 18 h in the presence of 0.5 mCi [^3^H]thymidine. The cells were harvested on glass-fiber filters, and the label uptake was determined using standard liquid scintillation techniques.

### Reverse transcription-polymerase chain reaction (RT-PCR) analysis of Olig1 expression

RT-PCR analysis was performed as previously reported [15]. Total RNA of the brain, spinal cord, lymph node, spleen and thymus was extracted with Isogen (Nippon Gene, Tokyo, Japan), treated with DNase (RQ1 RNase-free Dnase; Promega, Madison, WI, USA) and reverse-transcribed with Revertra ace (Toyobo, Osaka, Japan) to obtain cDNA. The primer set used was as follows: forward primer 5′-AGC CAG CCC TCA CTT GGA GAA CTG GGC CTG -3′, reverse primer 5′-TGC TGG GTA GCT CGC TGC AGG AGC TGC GCC -3′. The expected size of Olig1 product was about 300 bp.

### Immunoblot analysis

Immunoblotting were carried out as previously reported [28], [29]. Brains, optic nerves and spinal cords were freshly isolated from 6 week- and 15 week-old WT and Olig1^−/−^ mice, and then homogenized. Samples were separated on a sodium dodecyl sulfate-polyaclylamide gel electophoresis (SDS-PAGE) and subsequently transferred to an Immobilon-P filter (Millipore, Billerica, MA, USA). Membranes were incubated with an antibody against MOG (1∶1,000; Abcam, Cambridge, MA, USA), myelin basic protein (MBP; 1∶1,000; Santa Cruz, Santa Cruz, CA, USA), myelin-associated glycoprotein (MAG; 1∶1,000; Santa Cruz), myelin proteolipid protein (PLP; 1∶1,000; Abcam, Cambridge, MA), 2′,3′-cyclic nucleotide 3′-phosphodiesterase (CNPase; 1∶1,000; Sigma, St. Louis, MO), Olig2 (2 µg/ml; IBL, Gunma, Japan) or actin Ab-5 (1∶1,000; BD, Franklin Lakes, NJ, USA).

### Quantitative real-time PCR analysis of Olig2 in spinal cord

Quantitative RT-PCR of spinal cord cDNA was performed using the ABI 7500 fast real-time PCR system (Applied Biosystems, Foster City, CA) with SYBR Green PCR Master Mix (Applied Biosystems) as previously reported [15]. The primer probe pair for Olig2 was: forward primer 5′-CTG CTG GCG CGA AAC TAC AT-3′, reverse primer 5′-CGC TCA CCA GTC GCT TCA T-3′.

### Statistics

Data are presented as mean ± s.e.m. When statistical analyses are performed, Student's *t*-test was used to estimate the significance of the results.
